# NCOA3 is a selective co-activator of estrogen receptor α-mediated transactivation of PLAC1 in MCF-7 breast cancer cells

**DOI:** 10.1186/1471-2407-13-570

**Published:** 2013-12-04

**Authors:** Meike Wagner, Michael Koslowski, Claudia Paret, Marcus Schmidt, Özlem Türeci, Ugur Sahin

**Affiliations:** 1 Department of Internal Medicine III, Division of Translational and Experimental Oncology, University Medical Center, Johannes Gutenberg University, 55131 Mainz, Germany; 2 TRON-Translational Oncology gGmbH at the University Medical Center of the Johannes Gutenberg University, 55131 Mainz, Germany; 3 Department of Gynecology and Obstetrics, University Medical Center, Johannes Gutenberg University, 55131 Mainz, Germany; 4 Ganymed Pharmaceuticals AG, 55131 Mainz, Germany; 5 BioNTech AG, 55131 Mainz, Germany

**Keywords:** NCOA3, PLAC1, Estrogen-signaling, Breast cancer, Estrogen receptor α, Tumor antigen

## Abstract

**Background:**

The *placenta-specific 1* (*PLAC1)* gene encodes a membrane-associated protein which is selectively expressed in the placental syncytiotrophoblast and in murine fetal tissues during embryonic development. In contrast to its transcriptional repression in all other adult normal tissues, PLAC1 is frequently activated and highly expressed in a variety of human cancers, in particular breast cancer, where it associates with estrogen receptor α (ERα) positivity. In a previous study, we showed that ERα-signaling in breast cancer cells transactivates *PLAC1* expression in a non-classical pathway. As the members of the p160/nuclear receptor co-activator (NCOA) family, NCOA1, NCOA2 and NCOA3 are known to be overexpressed in breast cancer and essentially involved in estrogen-mediated cancer cell proliferation we asked if these proteins are involved in the ERα-mediated transactivation of *PLAC1* in breast cancer cells.

**Methods:**

Applying quantitative real-time RT-PCR (qRT-PCR), Western Blot analysis and chromatin immunoprecipitation, we analyzed the involvement of NCOA1, NCOA2, NCOA3 in the ERα-mediated transactivation of *PLAC1* in the breast cancer cell lines MCF-7 and SK-BR-3. RNAi-mediated silencing of NCOA3, qRT-PCR, Western blot analysis and ERα activation assays were used to examine the role of NCOA3 in the ERα-mediated regulation of PLAC1 in further detail. Transcript expression of *NCOA3* and *PLAC1* in 48 human breast cancer samples was examined by qRT-PCR and statistical analysis was performed using Student’s *t*-test.

**Results:**

We detected selective recruitment of NCOA3 but not NCOA1 or NCOA2 to the *PLAC1* promoter only in ERα-positive MCF-7 cells but not in ERα-negative SK-BR-3 breast cancer cells. In addition, we demonstrate that silencing of NCOA3 results in a remarkable decrease of PLAC1 expression levels in MCF-7 cells which cannot be restored by treatment with estradiol (E_2_). Moreover, significant higher transcript levels of *PLAC1* were found only in ERα-positive human breast cancer samples which also show a *NCOA3* overexpression.

**Conclusions:**

In this study, we identified NCOA3 as a selective co-activator of ERα-mediated transactivation of *PLAC1* in MCF-7 breast cancer cells. Our data introduce *PLAC1* as novel target gene of NCOA3 in breast cancer, supporting the important role of both factors in breast cancer biology.

## Background

Recently, we introduced *PLAC1* (*placenta-specific 1*) as a novel member of cancer-associated placental genes [1]. The *PLAC1* gene encodes a membrane-associated protein which is speculated to have a receptor-like function modulating specific cell-cell or ligand-receptor interactions unique to the maternal-placental interface [2,3]. Indeed, Jackman and coworkers recently disclosed *Plac1* as an essential factor for normal placental and embryonic development using a *Plac1* mutant mouse model [4]. In murine fetal tissues, Plac1 is expressed in several tissues, including brain, heart, kidney, liver, lung and intestine. *Plac1* knockout mice have an increased risk to develop a lethal hydrocephalus indicating that Plac1 plays a major role in brain development [5]. In adult normal tissues, expression of PLAC1 is strictly confined to differentiated cells of the placental syncytiotrophoblast, in which it is expressed throughout human gestation. In all other adult normal tissues, in contrast, PLAC1 underlies tight transcriptional repression. In a variety of human cancers, in particular breast cancer, PLAC1 is frequently activated and highly expressed [1]. Moreover, we found previously that PLAC1 is a critical factor for cancer cell proliferation, as silencing of *PLAC1* results in a pronounced G1 cell cycle arrest accompanied by decreased cyclin D1 expression and hypophosphorylation of AKT kinase. Tumor-promoting functions of PLAC1 can be antagonized by specific antibodies, thereby qualifying PLAC1 as promising candidate for targeted therapies of cancer.

The expression of PLAC1 is regulated by two distinct promoters, P1 and P2, separated by 105 kb. In human placenta and the human breast cancer cell line MCF-7, P2 is the preferred promoter, whereas P1 is preferentially used in the human choriocarcinoma cell lines BeWo and JAR [6]. By analysis of the promoter P2, we previously discovered in three different breast cancer cell lines that basal expression of PLAC1 is governed by the concerted action of the transcription factors SP1 and isoform 2 of CCAAT/enhancer binding protein β (C/EBPβ) that is selectively expressed in placental tissue and breast cancer cells [7]. Moreover, we showed that ERα-signaling in MCF-7 cells further transactivates *PLAC1* expression in a non-classical pathway that does not depend on the presence of estrogen-response elements (ERE), but rather by directly tethering activated ERα to DNA-bound SP1 and C/EBPβ-2. Accordingly, ERα-positive tumors display significantly higher PLAC1 expression levels compared with ERα-negative tumors [7].

Gene regulation by ERα requires the recruitment of a multitude of transcriptional co-regulators to the promoters of estrogen-responsive genes. Through interaction with these co-activator proteins, the activated receptor directs the assembly and stabilization of a pre-initiation complex that ultimately conducts the transcription of target genes. A key family of co-activators involved in the regulation of steroid receptor-mediated transcription is the p160/NCOA family [8], consisting of three members: nuclear receptor co-activator 1 (NCOA1) [9], NCOA2 [10] and NCOA3 [11], also known as steroid receptor co-activator 3 (SRC-3) and amplified in breast cancer 1 (AIB1) [12]. Gene amplification and overexpression of NCOA1, NCOA2 and NCOA3 in breast cancer has been described previously by several groups thus contributing to the development of cancer [8,12-21].

The prominent expression and function of PLAC1 particularly in breast cancer led our attention to this co-activator family. In the present study, we examined the involvement of p160/NCOA family members in the regulation of *PLAC1* in ERα-positive and -negative breast cancer cells and identified NCOA3 as a selective co-activator of ERα-mediated transactivation of *PLAC1* in ERα-positive MCF-7 breast cancer cells.

## Methods

### Tissues, cell lines and culture conditions

Breast cancer tissues were obtained as human surplus materials during routine diagnostic or therapeutic procedures and were stored at −80°C until use. The study was approved by the ethical review board of the medical association of Rhineland-Palatinate. Informed consent has been obtained and all clinical investigation has been conducted according to the principles expressed in the Declaration of Helsinki. Breast cancer cell lines SK-BR-3 and MCF-7 were obtained from American Type Culture Collection (ATCC) and cultured in Dulbecco’s modified Eagle’s medium (DMEM), 10% fetal calf serum (FCS). Prior to estradiol-treatment studies, MCF-7 cells were cultured in phenol-red free medium supplemented with 10% charcoal stripped FCS for 72 h. Cells were treated with 100 nM 17-β-estradiol (E_2_) (Sigma Aldrich) and/or with 5 μM of the complete estrogen receptor blocker ICI 182,780 (ICI) (Sigma Aldrich) for 12 h.

### Small interfering RNA duplexes and transfection

A pool of four different siRNA duplexes (Dharmacon) targeting *NCOA3* mRNA sequence (NM_006534) (siRNA 1 target sequence 5′-CAC AAU ACC UGC AAU AUA A-3′, siRNA 2 target sequence 5′-GAA AGG UUG UCA AUA UAG A-3′, siRNA 3 target sequence 5′-GAA GGU GUA UUC AGA GAU U-3′, siRNA 4 target sequence 5′-CGG AAA CAU UGU AUU UGU A-3′) or a mixture of two different siRNA duplexes (Qiagen) targeting *PLAC1* mRNA sequence (NM_021796) (siRNA 1 targeting nucleotides 342 to 362; target sequence 5′-CUC CAU GAG AGU AGC CAG CAA-3′ and siRNA 2 targeting nucleotides 670 to 690; target sequence 5′-CCG GUU CAG GAC AAA GTC CAA-3′) was used. As a control, a pool of four non-silencing (ns) siRNA duplexes (D-001810-10-05; Dharmacon) was used. MCF-7 cells were transfected with 5 nM siRNA duplexes using HiPerFect transfection reagent (Qiagen) and SK-BR-3 cells with 100 nM siRNA duplexes using DharmaFect 1 (Dharmacon) according to the respective manufacturer’s protocol. 48 h after transfection, cells were harvested for isolation of total RNA, subsequent cDNA synthesis and quantitative real-time RT-PCR or whole cells were lysed for western blot analysis.

### RNA-isolation, first-strand cDNA synthesis and quantitative real-time RT-PCR

Total RNA from cells or tissues was isolated using RNeasy Mini Kit (Qiagen) and subsequent conversion to cDNA was conducted using SuperScript II Kit (Invitrogen) according to the manufacturer’s instructions. Quantitative real-time RT-PCR was performed using the ABI PRISM 7300 sequence detection system and software (Applied Biosystems with QuantiTect SYBR green Kit (Qiagen)). The reactions were performed in duplicates in a 40 cycle PCR. After normalization to the housekeeping gene *HPRT1* (sense 5′-TGA CAC TGG CAA AAC AAT GCA-3′; antisense 5′-GGT CCT TTT CAC CAG CAA GCT-3′; 62°C annealing) the relative expression of *NCOA1* (sense 5′-GGC ATC AAT ATG AGA TCA GGC ATG-3′; antisense 5′-TTC CTA TCG CTC CTT GCT GCC A-3′; 62°C annealing), *NCOA2* (sense 5′-CTG GAG TAC CAA CAC AGG CAC-3′; antisense 5′-CTG TGC ATT TGC CTG GGG AAT CC-3′; 62°C annealing), *NCOA3* (sense 5′-GAA AGA GCA TTA TTG GAC CAG C-3′; antisense 5′-TGT CCC TGA AGA TGA AAG CC-3′; 60°C annealing) and *PLAC1* (sense 5′-AAA TTT GGC AGC TGC CTT CAC-3′; antisense 5′-TGA TGC CAC ATT CAG TAA CAC-3′; 60°C annealing) was quantified using ΔΔCt calculation. A calibrator of 16 corresponding to 40 (maximal number of cycles used in the PCR) minus the mean of the *HPRT1* values of the breast cancer tissue samples was used in this analysis.

### Western blot analysis

Whole cell lysates were prepared in 4X Laemmli sample buffer (250 mM Tris HCl, 34% glycerol, 8.2% SDS, 0.008% bromphenol blue) and supplemented with 5% β-mercaptoethanol. Extracts were subjected to SDS-PAGE and subsequently blotted onto nitrocellulose membrane (Whatman). Immunostaining was performed using mouse or rabbit monoclonal antibodies reactive against NCOA1 (128E7; Cell Signaling), NCOA3 (5E11; Cell Signaling), PLAC1 (22-3A-1; Ganymed Pharmaceuticals) and β-actin (AC-15, Sigma Aldrich) or using a polyclonal rabbit antibody reactive to NCOA2 (ab10491, Abcam). Detection of primary antibodies was performed with horseradish peroxidase-conjugated goat-anti-mouse or goat-anti-rabbit secondary antibodies (Jackson ImmunoReseach Laboratories). β-actin was used as a loading control.

### Chromatin immunoprecipitation (ChIP)

ChIP assays of MCF-7 and SK-BR-3 nuclear cell extracts were performed as previously described [7]. In short, ChIP was performed with chromatin prepared from MCF-7 or SK-BR-3 cells, which were either untreated or treated with 17-β-estradiol (E_2_). As additional controls, cells treated concomitantly with 17-β-estradiol and the estrogen receptor blocker ICI 182,780 or this compound alone were included. The purified DNA was analyzed by conventional PCR and quantitative real time PCR for the presence of the human *PLAC1* promoter fragment −348/-198 bp containing the C/EBPβ and SP1 elements (sense 5′-CAA CAG CAA GCA CTA CAA GTG-3′; antisense 5′-GAA GCT CAA CTC GGT GCA CTT GTT C-3′) and for a fragment −1219/-1064 bp upstream of the promoter as negative control (sense 5′-AAG CAC TTA GGA CAG CAT CTG-3′; antisense 5′-TGA ATG ATA CCT ACT GTC ATG) following immunoprecipitation with antibodies reactive to SP1 (ab13370), C/EBPβ-2 (ab32358), ERα (ab2746), NCOA1 (ab84), NCOA2 (ab9261), NCOA3 (ab2782), p300 (ab14984), pCAF (ab12188), AcH3 (ab12179), AcH4 (ab15823), TFIIB (ab12094) and RNA-Polymerase II (ab5131) (all from Abcam).

### Statistical analysis

Two-tailed Student’s *t*-test was used to analyze statistical significance of *NCOA3* compared to *NCOA1* and *NCOA2* transcript expression in MCF-7 and SK-BR-3 cells and *PLAC1* transcript expression in human breast cancer samples. *P*-values ≤ 0.05 (*) were considered statistically significant.

## Results

**Figure 1:**
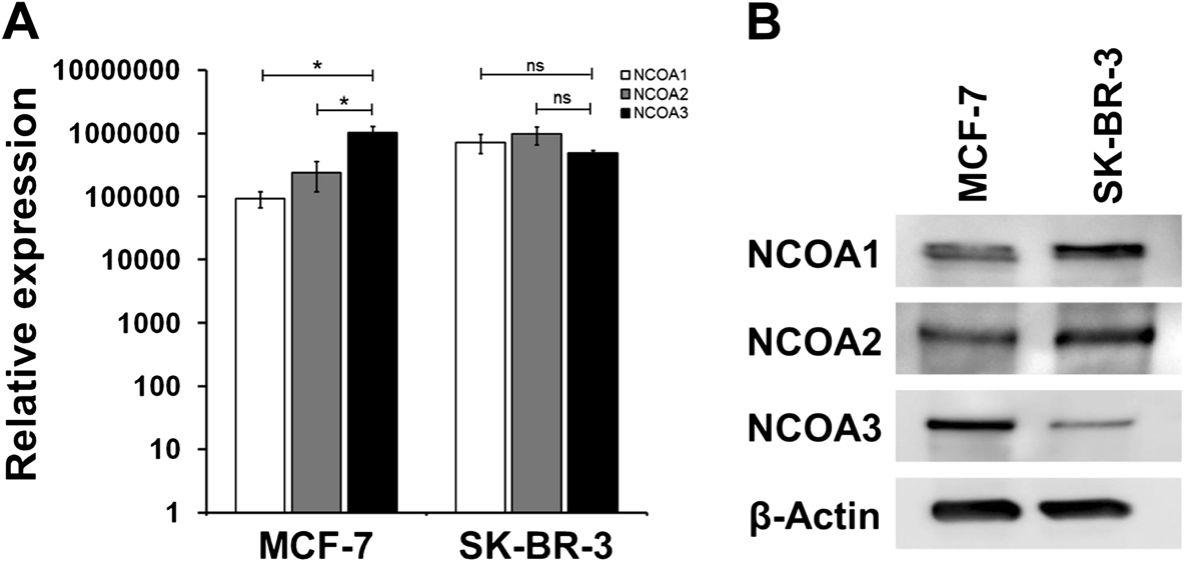
**Expression of NCOA1, 2 and 3 in ERα-positive MCF-7 and ERα-negative SK-BR-3 breast cancer cells. (A)** Real-time RT-PCR quantification of *NCOA1, 2 and 3* mRNA expression in MCF-7 and SK-BR-3 breast cancer cells. Shown are mean and standard deviation of three individual experiments. Statistical analysis of *NCOA3* mRNA expression compared to *NCOA1* and *2* were performed using two-tailed Student’s *t*-test. *P*-values ≤ 0.05 (*) were considered statistically significant. **(B)** Protein expression of NCOA1, 2 and 3 in MCF-7 and SK-BR-3 breast cancer cells was analyzed by Western blot. β-actin was used as a loading control.

In a first experiment, we analyzed expression of NCOA1, NCOA2 and NCOA3 in ERα-positive MCF-7 and ERα-negative SK-BR-3 breast cancer cells that both have been shown to express PLAC1 protein [1,7]. Expression of all three co-activators could be confirmed in both cell lines on transcript level by quantitative real-time RT-PCR and on protein level by Western blot analysis (Figure 1A, 1B). We found significantly higher expression of NCOA3 compared to NCOA1 and NCOA2 in MCF-7 cells, whereas in SK-BR-3 cells expression of NCOA3 was slightly reduced compared to the other family members.

**Figure 2:**
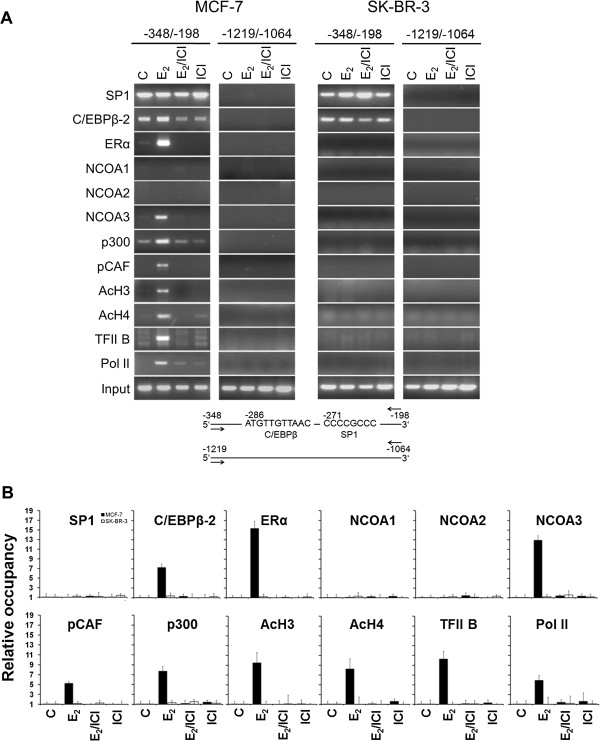
**Selective E**_**2**_**-dependent recruitment of NCOA3 to the endogenous *PLAC1 *promoter in ERα positive MCF-7 cells. (A)** Chromatin immunoprecipitation (ChIP) was performed with chromatin prepared from MCF-7 and SK-BR-3 cells which were either untreated (C), treated with 17-β-estradiol (E_2_) alone, with ICI alone or with both compounds (E_2_/ICI). The promoter region of *PLAC1* containing the C/EBPβ and SP1 elements (−348/-198) and a region upstream of the promoter (−1219/-1064) as negative control were analyzed by PCR following immunoprecipitation with the indicated antibodies. Amplification products from soluble chromatin prior to precipitation are shown as control (Input). **(B)** Quantitative analysis of the recruitment and occupancy shown in **(A)** determined by real-time RT-PCR. The results corrected by input are shown as fold increase compared to unstimulated cells used as a reference.

Next, we sought to determine if members of the p160/NCOA family participate in the formation of an estradiol (E_2_)-dependent pre-initiation complex on the endogenous *PLAC1* promoter. Chromatin immunoprecipitation (ChIP) assays were performed with nuclear extracts from untreated MCF-7 and SK-BR-3 cells or cells treated with 100 nM E_2_ in presence or absence of the ER-antagonist ICI 182,780 (ICI), which is known to efficiently inhibit the actions of endogenous and exogenous E_2_, or with ICI alone. We could confirm our recent data [7], showing that recruitment of C/EBPβ-2 and ERα to the *PLAC1* promoter was strongly and specifically induced by E_2_ in MCF-7 cells, whereas no apparent differences in the recruitment of SP1 in the presence or absence of E_2_ was observed (Figure 2A, 2B). Interestingly, we now detected recruitment of NCOA3, but not NCOA1 and NCOA2 to the complex by E_2_/ERα.

Full NCOA3 co-activator function also requires the recruitment of the histone acetyl transferases p300 and pCAF. Acetylation of histones by these factors modifies chromatin structure and provides a more accessible promoter environment for recruitment of components of the general transcriptional machinery [22]. Accordingly, we found specific recruitment of histone acetyl transferases p300 and pCAF, acetylated histones H3 and H4, as well as general transcription factor IIB (TFIIB) and RNA polymerase II (Pol II) to the *PLAC1* gene promoter upon E_2_-treatment. In contrast, in ERα-negative SK-BR-3 cells, formation of this pre-initiation complex by treatment with E_2_ could not be observed. These results indicate a selective involvement of NCOA3 in the assembly of an active E_2_/ERα-induced pre-initiation complex on the *PLAC1* promoter.

**Figure 3:**
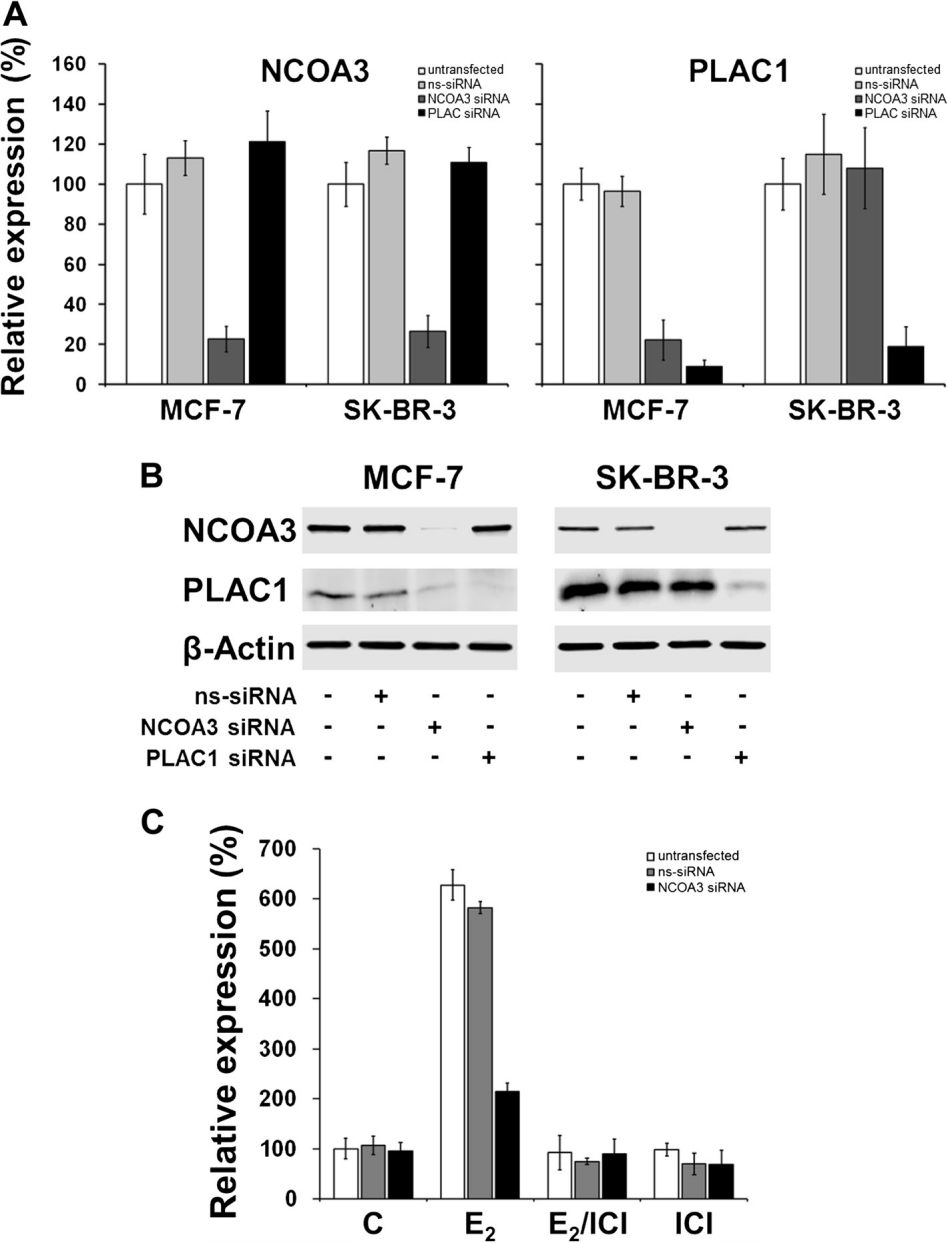
**ERα-mediated transactivation of PLAC1 is dependent on NCOA3. (A)** Quantitative real-time RT-PCR expression analysis of *NCOA3* and *PLAC1* in MCF-7 and SK-BR-3 cells 48 h after transfection with *NCOA3*- or *PLAC1*-specific siRNA duplexes. *ns-siRNA*, non-silencing siRNA. The results are shown as fold increase compared to untransfected cells used as a reference. **(B)** Protein expression of NCOA3 in MCF-7 and SK-BR3 cells 48 h after transfection with siRNA duplexes targeting *NCOA3* or *PLAC1* mRNA. Transfection of cells with *PLAC1*-specific siRNA was conducted to verify that knockdown of NCOA3 specifically affects PLAC1 mRNA and protein level. **(C)** Quantitative real-time RT-PCR analysis of *PLAC1* expression in E_2_-depleted (72 h) MCF-7 cells in response to treatment with 100 nM E_2_, no E_2_ (C), or E_2_ and 5 μM ICI (E2/ICI), or ICI alone for 12 h. 48 h prior to treatment, cells were transfected with siRNA duplexes targeting *NCOA3*. Shown are mean and standard deviation of two independent experiments.

To directly assess the impact of NCOA3 on PLAC1 transcript and protein expression, we silenced *NCOA3* in MCF-7 and SK-BR-3 cells by transient transfection with specific siRNA duplexes. Transcript levels of *NCOA3* were specifically reduced by 80% compared to non-transfected cells and cells transfected with non-silencing control siRNA duplexes in both cell lines (Figure 3A). Strikingly, silencing of *NCOA3* resulted in a concomitant loss of *PLAC1* expression only in ERα-positive MCF-7 cells but not in ERα-negative SK-BR-3 cells. These findings could be confirmed on protein level by Western blot analysis (Figure 3B). Noteworthy, silencing of *NCOA3* in MCF-7 cells did not result in complete loss of PLAC1 expression, as basal expression of PLAC1 in breast cancer is not dependent on ERα-signaling but rather on the presence of SP1 and C/EBPβ-2 [7]. The importance of NCOA3 for E_2_-mediated transactivation of *PLAC1* was further verified in E_2_-depleted MCF-7 cells transfected with siRNA duplexes (Figure 3C). Exposure of these cells to 100 nM E_2_ induced *PLAC1* expression only in non-transfected cells and cells transfected with non-silencing siRNA duplexes. In contrast, *PLAC1* transactivation was nearly abolished in cells lacking NCOA3, further confirming the important function of NCOA3 for E_2_/ERα-induced transactivation of *PLAC1* in breast cancer cells.

**Figure 4:**
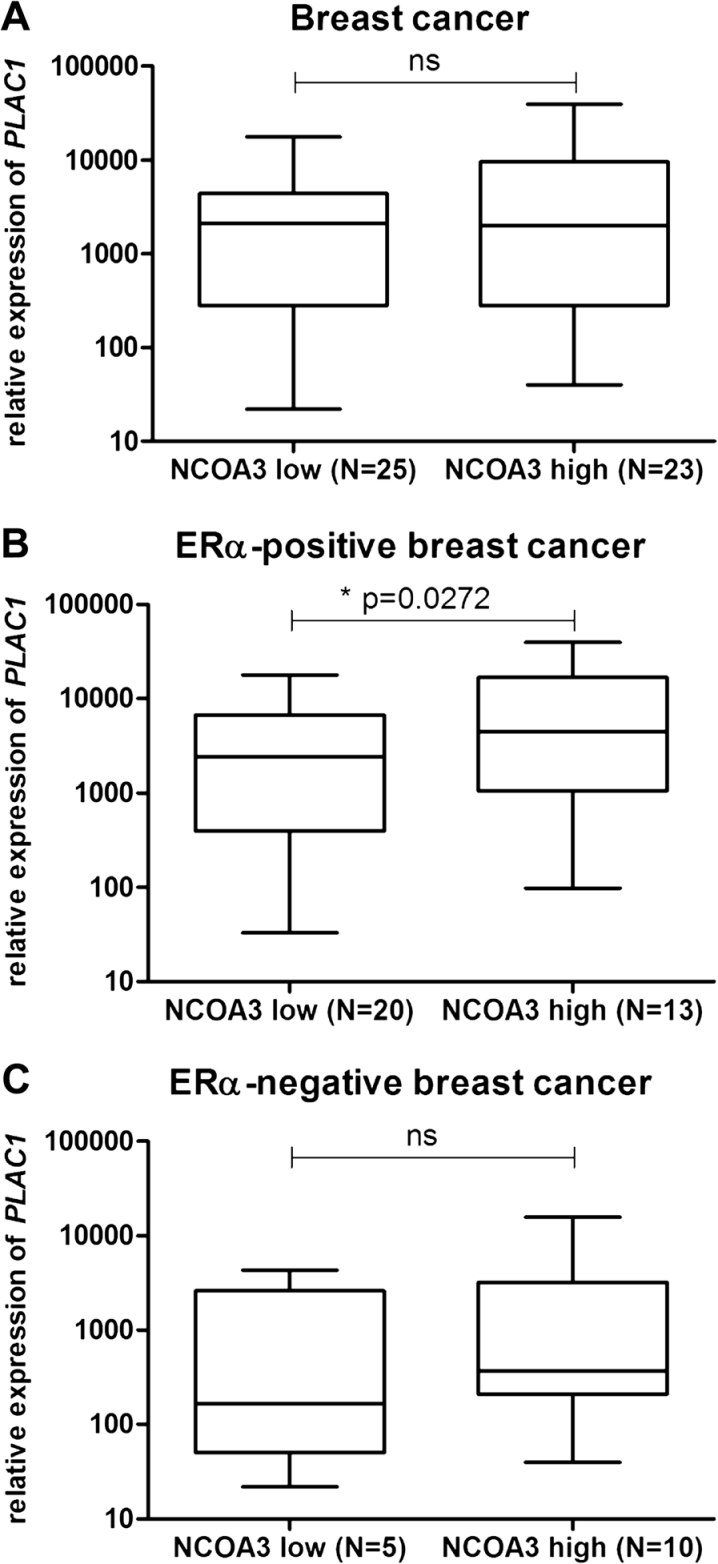
**mRNA expression of *PLAC1 *is elevated in *NCOA3 *overexpressing and ERα-positive human breast cancer samples. (A)***NCOA3* and *PLAC1* mRNA expression was examined by quantitative real-time RT-PCR in 48 human breast cancer samples. Relative expression of *NCOA3* below 100 000 were considered to be ‘*NCOA3* low’ (N = 25), whereas relative expression of *NCOA3* over 100 000 were considered to be ‘*NCOA3* high’ (N = 23). **(B)** Evaluation of *PLAC1* expression in ERα-positive breast cancer samples with ‘*NCOA3* low’ (N = 20) or ‘*NCOA3* high’ (N = 13) status. **(C)** Evaluation of *PLAC1* expression in ERα-negative breast cancer samples with ‘*NCOA3* low’ (N = 5) or ‘*NCOA3* high’ (N = 10) status. Statistical analysis was performed using two-tailed Student’s *t*-test. *P* ≤ 0.05 (*) was considered statistically significant.

To consolidate our data *in vivo*, we analyzed expression of *PLAC1* and *NCOA3* transcripts in 48 human primary breast cancer tissue samples. Since overexpression of NCOA3 in breast cancer has been shown to be associated with clinical parameters [14,23], we were interested if high *PLAC1* expression is associated with *NCOA3* overexpression. Therefore, we subdivided the 48 breast cancer samples into two groups depending on *NCOA3* expression levels. Relative expression of *NCOA3* below 100 000 (in contrast, relative expression of *NCOA3* in normal breast tissue is about 10 000, data not shown) were considered to be ‘*NCOA3* low’ (N = 25), whereas relative expression of *NCOA3* over 100 000 were considered to be ‘*NCOA3* high’ (N = 23). While no difference in *PLAC1* expression was found between *NCOA3* low and *NCOA3* high expressing breast cancer tissues regardless of the ER-status or in the ERα-negative samples, a significant higher expression of *PLAC1* in the *NCOA3* high expressing breast cancer samples was detected exclusively in ERα-positive breast cancer samples (Figure 4).

## Discussion

In this study, we have characterized the role of the p160/NCOA family of co-activators in the regulation of *PLAC1* in breast cancer cells. Our data show that NCOA3 but not NCOA1 or NCOA2 is selectively recruited to the *PLAC1* promoter upon E_2_-treatment. Silencing of *NCOA3* results in loss of *PLAC1* transactivation after E_2_-stimulation only in ERα-positive MCF-7 but not in ERα-negative SK-BR-3 breast cancer cells. Moreover, we observed a significant correlation of *PLAC1* expression and NCOA3 overexpression in a cohort of ERα-positive breast cancer patients.

The members of the p160/NCOA family of co-activators function in a similar fashion to stimulate the transcription of nuclear receptor target genes. However, despite their significant homology in structure and sequence NCOA1, NCOA2 and NCOA3 exhibit unique, non-redundant functions in different cell types and tissues [8,21,24]. In human breast cancer, NCOA3 is amplified and overexpressed at transcript and protein level in a significant portion of tumors [12,20,25] and its particular relevance is underlined from the induction of spontaneous mammary tumors in mice overexpressing NCOA3 in mammary epithelial cells [26]. This is supported by studies showing that NCOA3 can stimulate the growth of breast cancer cell lines through estrogen-independent and estrogen-dependent mechanisms. In fact, NCOA3 promotes the transcriptional activity of several transcription factors, including E2F-1, RAR, NFκB, and STAT6 [27-30].

In ER-positive breast cancers, NCOA3 is required for maximal activity of ERα and other hormone receptors [31,32]. Changes in NCOA3 expression influence ERα-dependent gene expression and consequently modulate cellular processes that promote cancer such as proliferation, invasion and cell motility [33,34]. Indeed, depletion of NCOA3 protein in MCF-7 cells results in decreased E_2_-stimulated proliferation *in vitro* and a decreased growth of MCF-7 cells in xenografts in mice. This function of NCOA3 is mediated through downstream effectors like cyclin D1, which plays a pivotal role in estrogen-dependent proliferation [35]. Interestingly, we found previously that PLAC1 is a critical factor for breast cancer progression, as RNAi-mediated silencing of PLAC1 in MCF-7 and BT-549 breast cancer cells results in decreased cyclin D1 levels and induces a G1-S cell cycle block with nearly complete abrogation of proliferation [1]. Thus our data introduce PLAC1 as a possible downstream effector of NCOA3 mediated actions in ERα-positive breast cancer cells. However, further experiments are required to also show a functional correlation between E_2_-induced breast cancer cell proliferation and *PLAC1* expression.

Noteworthy, NCOA3 overexpression in breast cancer correlates with larger tumor size, higher tumor grade, as well as with the expression of ERBB2 [8]. Furthermore, high levels of NCOA3 are associated with resistance to therapy and a shorter disease free survival in patients with ERα-positive tumors treated with the selective ER-modulator Tamoxifen. Based on these observations NCOA3 has been under investigation as a target for treatment of breast cancer [36]. Since we detect a significant correlation between *NCOA3* overexpression and *PLAC1* expression in ERα-positive breast cancer patients this adds to the relevance of understanding the role of PLAC1 in the NCOA3/ERα-signaling pathway and might open new therapeutic concepts for breast cancer.

The complex expression pattern of PLAC1 in placenta and cancer is regulated by two distinct promoters. In this study, we demonstrate explicit involvement of NCOA3 in the formation of an E_2_-dependent pre-initiation complex at the *PLAC1* P2 promoter. The P2 promoter transcript predominates in placenta and MCF-7 breast cancer cells. However, both promoters are active to some extent in placenta as well several other cancer-derived cell lines and can be both activated by retinoid X receptor alpha (RXRα) in conjunction with liver X receptor alpha (LXR) [6]. RXRα functions as homodimers or as heterodimers with other nuclear receptors including peroxisome proliferator-activated receptor (PPAR) isoforms [23]. Indeed, it was shown that activation of PPARδ in the mouse mammary epithelium results in the appearance of estrogen receptor- and progesterone receptor-positive and ErbB2-negative infiltrating ductal carcinomas which are associated with an up-regulation of Plac1 [37]. This suggests a function of PPARδ in the regulation of Plac1 but remains to be proven.

Furthermore, very recently, involvement of TP53, RB and NCOA2 in regulation of *PLAC1* at promoter P1 was demonstrated in SV40 transduced primary fibroblasts. While expression of TP53 represses *PLAC1* promoter P1, RB potentiated *PLAC1* transcription in conjunction with NCOA2, particularly when RXRα and LXR agonists were present [38]. These results suggest that NCOA2 and NCOA3 act under different circumstances as activators of *PLAC1* from promoter P1 or P2 respectively. In which extend NCOA3 can act at the promoter P1 for example in placenta remains to be analyzed. Intriguingly, NCOA3 can act as a co-activator also of PPARs [23] and was found to inhibit TP53 in breast cancer cells [39] implicating a broader involvement of NCOA3 in regulation of *PLAC1*. Thus, additional studies are required to dissect the complex regulation of *PLAC1* in placenta and cancer tissues.

## Conclusions

In conclusion, our results shed further light on the composition of the E_2_/ERα-mediated transactivation complex at the PLAC1 promoter in breast cancer cells. Our results show that NCOA3 is necessary for E_2_-dependent transactivation of *PLAC1* in an ERα-dependent manner. Moreover, we demonstrate a relevant correlation of *PLAC1* expression and *NCOA3* overexpression in human breast cancer tissues restricted to the ERα-positive samples. Our findings provide the basis to further dissect the role of PLAC1 in the ERα-signaling pathway in breast cancer and suggest PLAC1 as a novel target gene of NCOA3 in ERα-positive MCF-7 breast cancer cells supporting the role of both factors in breast cancer biology. The functional relationship between PLAC1 and NCOA3 as well as the correlation analysis of *PLAC1* expression with various clinical parameters like tumor size, overall and disease-free survival as well as resistance to therapy will be subjects of further studies.

## Competing interests

US, ÖT and MK are holding patent applications on PLAC1 as therapeutic target for antibody-based cancer immunotherapy. ÖT is CEO/CSO of Ganymed Pharmaceuticals AG. US is managing director (science and research) of TRON and founder of BioNTech AG. MW, CP and MS declare that they have no competing interests.

## Authors’ contributions

MW and MK carried out the experiments. MW, MK, CP and US designed the study. MW, MK and CP wrote the manuscript. MS provided the breast cancer tissues. ÖT provided the anti-PLAC1 antibody. ÖT and US contributed to the manuscript draft. All authors have read and approved the final manuscript.

## Pre-publication history

The pre-publication history for this paper can be accessed here:

http://www.biomedcentral.com/1471-2407/13/570/prepub
